# Frequency of Infection during Fever Episodes among Long-Term Care Residents

**DOI:** 10.4172/2167-7182.1000467

**Published:** 2018-04-09

**Authors:** Rupak Datta, Sonali Advani, Andrea Rink, Luann Bianco, Peter H. Van Ness, Vincent Quagliarello, Manisha Juthani-Mehta

**Affiliations:** 1 Yale School of Medicine, Department of Internal Medicine, Section of Infectious Diseases, New Haven, CT USA; 2 Yale School of Medicine, Department of Internal Medicine, Section of Geriatrics, New Haven, CT USA

**Keywords:** Fever, Infection, Long-term, Care, Residents

## Abstract

**Background:**

Empirical data regarding the frequency of infection during fever episodes among women in long-term care facilities are lacking.

**Methods:**

We conducted a case-series analysis of women long-term care residents enrolled in a randomized trial evaluating cranberry capsules to reduce bacteriuria plus pyuria across twenty-one long-term care facilities in CT, USA. Fever episodes identified during adverse event surveillance were assessed using established guidelines for older adults. Among fever episodes, infections were classified using standardized infection surveillance definitions in long-term care residents.

**Results:**

We identified 123 fever episodes among 80 women long-term care residents. Median age was 88 years (range, 65–101), and 81% (N=65) had dementia. Among 123 fever episodes, 79 (64%) met criteria for 86 total infections (lower respiratory tract, N=43; pneumonia, N=27; gastroenteritis, N=9; urinary tract, N=7).

**Conclusion:**

Data from this study suggest that approximately two-thirds of fever episodes involve infection among women in long-term care facilities. These data may guide provider assessments of fever in older adult women in long-term care facilities.

## Introduction

Comorbid conditions affecting older adults may preclude reliable clinical assessment of infection [1]. Consequently, infection diagnoses often rely on vital signs [2]. However, fever interpretation among older adults is complicated by lower baseline temperatures, blunted host immune responses, and contributing non-infectious causes [3–5]. The importance of accurate fever interpretation is underscored by widespread antimicrobial overuse in long-term care settings [6]. We sought to provide empirical data regarding the frequency of infectious causes for fever episodes among women in long-term care facilities.

## Methods

We conducted a case series analysis of all adverse events in women residents enrolled in a randomized trial evaluating cranberry capsules to reduce bacteriuria plus pyuria across 21 long-term care facilities between August 2012 and October 2015 [7]. Fever episodes that were identified during adverse event surveillance were subsequently evaluated using Infectious Diseases Society of America (IDSA) guidelines for older adults [8]. The Yale University Human Investigation Committee approved this study.

For all participants, we recorded demographics, comorbidities, and medications. Fever episodes were evaluated in a stepwise manner. Initially, we assessed whether temperatures exceeded 100 F. Among temperatures <100 F, we assessed whether two oral temperatures exceeded 99 F during the adverse event and subsequently whether temperatures were 2 F above baseline (i.e., last temperature documented prior to adverse event). Based on this hierarchy, fever episodes were identified and plotted against baseline temperatures. For all fever episodes, we identified related hospitalizations, deaths, diagnostic tests, infections and antimicrobials. Infections were classified using surveillance definitions [9]. Antimicrobial-days were described among fever episodes with and without infection.

## Results

Among 3830 total adverse events, we identified 123 (3%) fever episodes among 80 women participants. Median age was 88 years (range, 65–101), and 94% (N=75) were white. Of these 80 participants, 81% (N=65) had dementia, 34% (N=27) had congestive heart failure, and 20% (N=16) had daily bladder incontinence. Median number of medications was 11 (range, 3–20).

**Figure 1:**
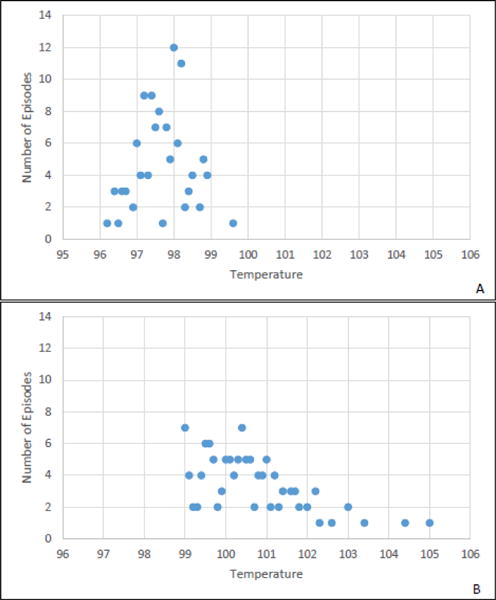
Baseline (A) and febrile (B) temperature distribution among 123 fever episodes. Fever episodes were identified from 80 women long-term care residents enrolled in a randomized trial. Baseline temperature was defined as the last routine temperature documented prior to the adverse event. All residents have both baseline and febrile temperatures depicted in the Figure 1. Each dot may represent more than one resident.

Among 123 fever episodes, 79 (64%) met criteria for 86 total infections (lower respiratory tract, N=43; pneumonia, N=27; gastroenteritis, N=9; urinary tract, N=7). There were no *Clostridium difficile* infections. Infections occurred among fever episodes managed with (N=60/83, 72%) and without (N=19/40, 48%) administration of antimicrobials (Table 1). Figure 1 shows the baseline and febrile temperature distribution. Median antimicrobial-days varied between fever episodes with (6 days, range 0–36) versus without (1 day, range 1–98) infection.

## Discussion

Among fever episodes in women long-term care residents, we show that approximately two-thirds met criteria for infection. Among fever episodes managed with empiric antimicrobials, the majority met surveillance criteria for infection. These findings have implications for providers managing long-term care residents admitted with fever and provide empiric data supporting the use of consensus-derived definitions for fever and infection.

Our work highlights the complementary roles of antimicrobial and diagnostic stewardship. Whereas most fever episodes were managed judiciously with antimicrobials, with prompt discontinuation when infection was excluded, diagnostic testing may be inappropriate and occasionally excessive. In our sample, there were seven-fold more urine cultures ordered than urinary tract infections identified. Evidence suggests multifaceted interventions reduce inappropriate urine cultures in hospitals [10]. The data from this study, however limited, suggest that similar work needs to be conducted in long-term care facilities.

Our study has limitations. First, it may lack generalizability as only women were enrolled. However, our study builds upon data evaluating the association between body temperature and infection limited to older adults receiving antimicrobials [11]. Second, this was an observational secondary analysis, and diagnostic testing for infection was up to physician discretion. Third, temperature assessments were subject to random and unavoidable measurement error which may have caused fever misclassification. However, fever misclassification was likely non-differential with regard to infection, and this limitation has been described in prior reports [1,3,11]. Finally, we did not collect data on medications that may have masked fever.

## Conclusion

We show that approximately two-thirds of fever episodes involve infection among women in long-term care facilities when applying IDSA fever guidelines and established infection surveillance definitions. These data may guide provider assessments of fever in older women in long-term care facilities.

## Figures and Tables

**Table 1:** Descriptive characteristics of 123 fever episodes among 80 women long-term care residents participating in a randomized trial.

Characteristic	No. (%)
**Infectious diseases Society of America criteria for fever**	
Single temperature >100 F	77 (63%)
Repeated temperatures >99 F	28 (23%)
Temperature >2 F above baseline	18 (15%)
**Diagnostic tests ordered**	
Chest X-ray	65 (53%)
Urine culture	49 (40%)
Stool culture	1 (1%)
**Antimicrobial therapy**	
Administered	83 (67%)
Not Administered	40 (33%)
**Severity**	
Hospitalization	23 (19%)
Death	9 (7%)
